# Bacterial diversity obtained by culturable approaches in the gut of *Glossina pallidipes* population from a non sleeping sickness focus in Tanzania: preliminary results

**DOI:** 10.1186/s12866-018-1288-3

**Published:** 2018-11-23

**Authors:** Imna Malele, Hamis Nyingilili, Eugen Lyaruu, Marc Tauzin, B. Bernard Ollivier, Jean-Luc Cayol, Marie-Laure Fardeau, Anne Geiger

**Affiliations:** 1 Molecular Department, Vector and Vector Borne Diseases Institute, Majani Mapana, Off Korogwe Road, Box 1026, Tanga, Tanzania; 2 USC1342 INRA, UMR113 IRD-CIRAD-SupAgro-UM2, Symbioses Tropicales et Méditerranéennes, Campus de Baillarguet, TA A-82/J, F-34398 Montpellier Cedex 5, France; 3 Aix-Marseille UniversitéUniversité du Sud Toulon-Var, CNRS/INSU, IRD, Mediterranean Institute of Oceanography (MIO), UM 110, F-13288 Marseille cedex 09, France; 4 UMR 177, IRD-CIRAD, CIRAD TA A-17/G, Campus International de Baillarguet, 34398 Montpellier Cedex 5, France

**Keywords:** Facultative anaerobes, Aerobes, Microaerobes, Bacterial diversity, Gut, Sleeping sickness, *Glossina pallidipes*, Tanzania

## Abstract

**Background:**

*Glossina pallidipes* is a haematophagous insect that serves as a cyclic transmitter of trypanosomes causing African Trypanosomiasis (AT). To fully assess the role of *G. pallidipes* in the epidemiology of AT, especially the human form of the disease (HAT), it is essential to know the microbial diversity inhabiting the gut of natural fly populations. This study aimed to examine the diversity of *G. pallidipes* fly gut bacteria by culture-dependent approaches.

**Results:**

113 bacterial isolates were obtained from aerobic and anaerobic microorganisms originating from the gut of *G. pallidipes*. 16S rDNA of each isolate was PCR amplified and sequenced. The overall majority of identified bacteria belonged in descending order to the *Firmicutes* (86.6%), *Actinobacteria* (7.6%), *Proteobacteria* (5.5%)and *Bacteroidetes* (0.3%). Diversity of *Firmicutes* was found higher when enrichments and isolation were performed under anaerobic conditions than aerobic ones. Experiments conducted in the absence of oxygen (anaerobiosis) led to the isolation of bacteria pertaining to four phyla (83% *Firmicutes*, 15% *Actinobacteria*, 1% *Proteobacteria* and 0.5% *Bacteroidetes*, whereas those conducted in the presence of oxygen (aerobiosis) led to the isolation of bacteria affiliated to two phyla only (90% *Firmicutes* and 10% *Proteobacteria*). Phylogenetic analyses placed these isolates into 11 genera namely *Bacillus, Acinetobacter, Mesorhizobium, Paracoccus, Microbacterium, Micrococcus, Arthrobacter, Corynobacterium, Curtobacterium, Vagococcus* and *Dietzia* spp.which are known to be either facultative anaerobes, aerobes, or even microaerobes.

**Conclusion:**

This study shows that *G. pallidipes* fly gut is an environmental reservoir for a vast number of bacterial species, which are likely to be important for ecological microbial well being of the fly and possibly on differing vectorial competence and refractoriness against AT epidemiology.

**Electronic supplementary material:**

The online version of this article (10.1186/s12866-018-1288-3) contains supplementary material, which is available to authorized users.

## Background

Human African Trypanosomiasis (HAT) is transmitted by tsetse flies which belong to the genus *Glossina*. To be transmitted, the parasite (trypanosome) must first be established in the fly midgut, after an infective blood meal, and then mature in the salivary glands or mouthparts, depending on the trypanosome species [1, 2]. The success of the establishment and the maturation of trypanosomes play a key role in the disease transmission cycle. However the capacity of the fly to be infected and transmit trypanosomes, depends on several factors such as the tsetse fly species, the genetic variability within a given species, and the presence of the symbiotic microorganisms in the fly. The factors are important and influence the vector competence of tsetse flies [3].

It has been documented by Soumana et al. [4], Lindh & Lehane, [5] that tsetse midguts contain various microorganisms which include pathogens and others which may be useful to the fly. They include symbionts (e.g. *Sodalis, Wigglesworthia* and *Wolbachia* spp) as well as the *salivary gland hyperplasia virus* [6] and the parasitic nematodes (e.g. *Hexamermis glossinae*) [7]. Tsetse flies are highly depended on their microbial flora for providing nutrients that are not supplied by their restricted diet of vertebrate blood. In recent years, there has been an increased research interest on midgut microbial flora and their likely role to be played in the refractoriness of tsetse flies and in the epidemiology of African Trypanosomiasis. It was shown that the midgut of tsetse flies contained a diversity of microorganisms depending both on the tsetse species or sub species and the geographic origin of the flies [8, 9]. Further research on bacteria inhabiting four fly species namely *Glossina palpalis palpalis, G*. *pallicera, G. nigrofusca* and *G. caliginea* showed the occurrence of bacteria belonging to *Proteobacteria, Firmicutes,* and *Bacteroidetes* phyla [10]. Phylogenetic analyses basing on 16S rNA gene sequences revealed that they belongedto the genera *Acinetobacter, Enterobacter, Enterococcus, Providencia, Sphingobacterium, Chryseobacterim, Lactococcus, Staphylococcus,* and *Pseudomonas* [10]. Other studies on tsetse collected from East Africa (Kenya) showed the dominance of bacteria within the *Firmicutes* and especially those belonging to the genus *Bacillus*. Others were members of the *Actinobacteria, Beta –* and *Gammaproteobacteria* [5, 10]. Here we report on the culturable diversity of bacteria from the gut of *Glossina pallidipes* species collected from the non HAT (non sleeping sickness) area along the coastal area of Tanzania, Tanga region.

## Methods

### Description of the tsetse species, *Glossina pallidipes*

*Glossina pallidipes* is one of the tsetse species which transmits African Trypanosomiasis. In Tanzania, *G. pallidipes* is widely distributed, hence of economic importance in the epidemiology of African Trypanosomiasis. The disease is a stumbling block for diversification of agricultural activities as well as socio economic well being of rural areas. *G. pallidipes* occurs in all belts of the country covering those areas that are human African trypanosomisasis active foci, silent foci as well as in the areas where the disease has never been recorded.

### Trapping of tsetse species

Tsetse flies were trapped using 6 biconical traps [11] baited with acetone, during the month of October 2014 when it is normally hot and humid with short rains in the area; Temperatures and the relative humidity of 25 – 29 °C and 76–84% respectively. Collected flies were sampled from a non HAT area (site) Mgambo, Kabuku ward in Handeni (Tanga region) district and were transported to the laboratory for sorting them into species and only non teneral were selected using the tsetse identification manual [12] into species.

*Glossina pallidipes* was the only species trapped hence dissection for midgut collection was restricted from this tsetse specie.

### Microbial isolation, PCR amplification and sequencing

78 live *G. pallidipes* were dissected and midguts removed. Dissection was carried out after flies had been surface sterilized (once with 5% sodium hypochlorite and twice with 70% ethanol). Random selection was made to include both males and female non teneral tsetse flies. The midgut of each fly was sterilely dissected under a hood and ground with sterilized pestle. 40 midguts were cultured under aerobic condition and 38 midguts were cultured under anaerobic conditions using roll tubes and culture media as already reported by Geiger et al. [8, 10]. The gas phase of roll tubes contained air for aerobic conditions, while that of roll tubes prepared under anaerobic condition contained CO_2_. Experiments were stopped after 4 days incubation at 26 °C (room temperature). When positive growth was obtained in liquid culture media after three to seven days as observed by the increase of optical density, they were serially diluted in the same culture conditions with the aim to obtain axenic cultures. Culturing was one gut per tube. For this purpose, the last positive serial dilutions were streaked (from each gut) onto solid medium using Petri dishes, for aerobes (Luria Berthani /agar medium) and for anaerobes (Mitsuhashi-Maramorosch medium/blood/bovine foetal serum /agar). After obtaining individual colonies, the process of purification was undertaken as previously described [10]. In some cultures, more than one colony were picked per fly gut, but picking of individual colonies was based on morphology and care was made to ensure that all colonies from all fly midguts were included for further analysis. A total of 113 bacteria colonies were picked for further analysis and care was made to ensure bacteria colonies picked were recorded in order to trace the fly number and thus fly midgut; and whether the initial culture was by aerobic or anaerobic condition. The 16S rRNA gene of each of the 113 isolates was amplified using a PCR reaction as described by Geiger et al.*,* [10]. The PCR products for all bacteria colonies were sent to Bioneer (South Korea) for sequencing using three primers F1(5′-CTC-CTA-CGG-GAG-GCA-GCA-G-3′), Fd1 (5′-AGA GTT TGA TCC TGG CTC AG-3′) and Rd1 (5′-AAG GAG GTG ATC CAG CC-3′). The amplification was done using F1 and Rd1 which produces a fragment of about 1400 bp. The primers F1, Fd1 and Rd1 were again used for sequencing a fragment of about 600-800 bp each.

Obtained sequences were blast searched on NCBI databases and phylogenetic trees [13] and assembled using the PHYML program [14]. The blast search results and the phylogenetic trees allowed the identification of bacteria reported in the study. The datasets used and/or analysed during the current study are available from the publically available repository i.e. *“**https://dataverse.harvard.edu/**“.*

## Results

A total of 91 isolates cultivated under anaerobic culture conditions were obtained from 38 midguts and generated 88 sequences (3 isolates generated no sequences from this culture); while only 25 sequences were obtained after growth under aerobic conditions from 40 midguts. The largest groups of bacteria isolated from *G. pallidipes* from this coastal area belonged to the *Firmicutes* which accounted for 87% of total bacteria; followed by *Actinobacteria* 7.7%; *Proteobacteria* (5.5%) and *Bacteroidetes* 0.3% (Table 1).

**Table 1:** Prevalence of occurrence of different bacteria phylum per isolation conditions

Phylum	Prevalence of occurrences (%)		Overall prevalence (%)
	Anaerobic	Aerobic	
*Firmicutes*	83	90	87
*Proteobacteria*	1	10	5.5
*Actinobacteria*	15	0	7.5
*Bacteroidets*	1	0	0.5

A more diverse bacterial population was obtained under anaerobic conditions as compared to aerobic ones (Tables 1 and 2). Members of four bacterial phyla were isolated from enrichments performed under anaerobiosis (83% for *Firmicutes*, 15% *Actinobacteria*, 1% *Proteobacteria* and 0.5% *Bacteroidetes*) where as members of only two phyla were isolated under aerobiosis (90% *Firmicutes* and 10% *Proteobacteria*). *Firmicutes* and *Proteobacteria* were recorded in both culture conditions. It is noteworthy that *Actinobacteria* and *Bacteroidetes* isolated were only retrieved under anaerobiosis (Table 1).

**Table 2:** Taxonomic positioning of bacteria at the phylum and genus level isolated under aerobic and anaerobic conditions

Phylum	Genera	Counts of bacteria spp. isolated under anaerobic conditions (*n* = 88)	Counts of bacteria isolated under aerobic conditions (*n* = 25)
*Firmicutes*	*Bacillus*	3	0
	*Vagococcus*	0	1
	*Enterococcus*	0	1
	*Staphylococcus*	26	8
*Proteobacteria*	*Acinetobacter*	1	0
	*Mesorhizobium*	0	1
	*Paracoccus*	1	0
	*Psychrobacter*	1	1
*Actinobacteria*	*Microbacterium*	4	0
	*Micrococcus*	3	0
	*Arthrobacter*	4	0
	*Corynebacterium*	1	0
	*Curtobacterium*	1	0
	*Dietzia*	1	0
*Bacteroidetes*	*Flavobacterium*	1	0

### Diversity of bacteria in individual fly gut

**Fig. 1:**
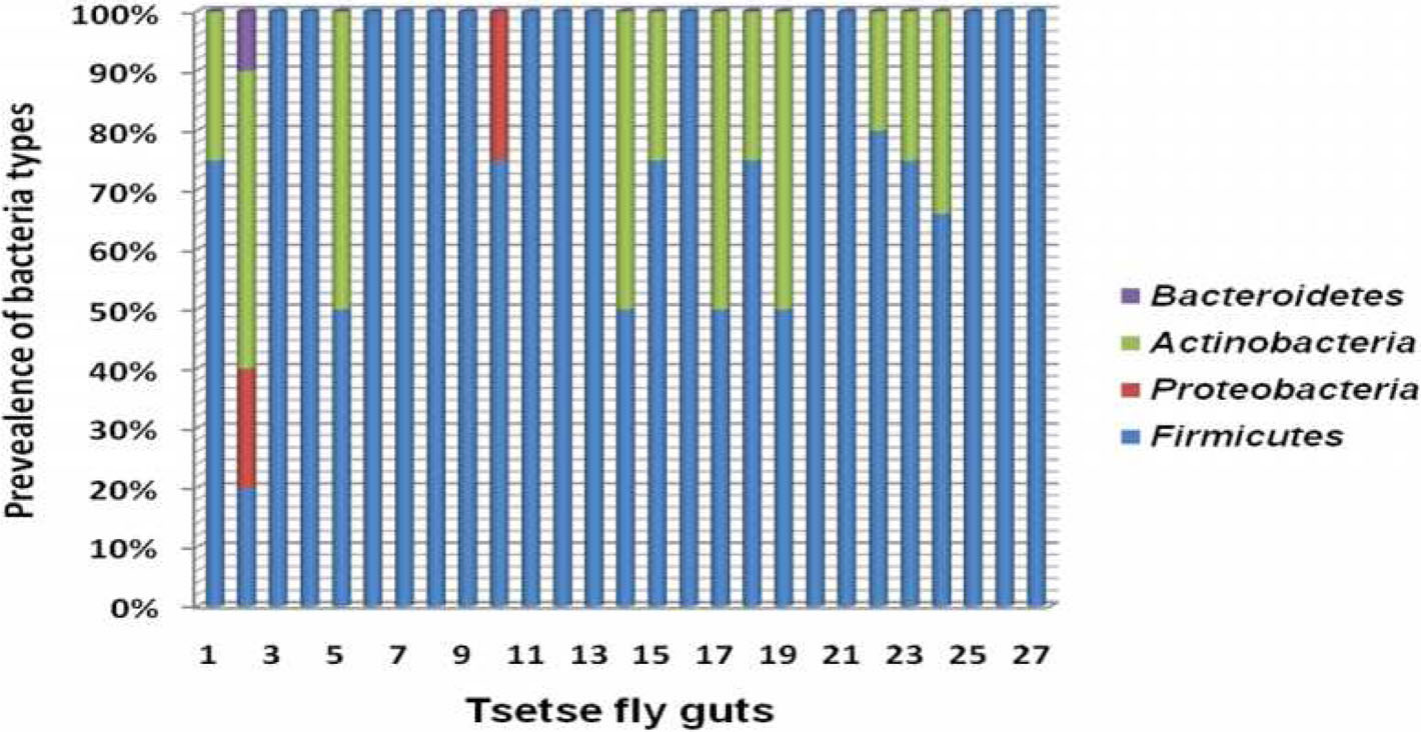
Prevalence of bacterial types obtained per phyla per fly midgut under anaerobic conditions

When assessing the bacterial diversity per individual fly gut, our results showed that it was common to find more than one bacterial phylum inhabiting the same fly gut. Colony bacteria were numbered according to fly gut number and the sequences generated were according to the bacteria numbers which directly related to the fly number. For instance, *Actinobacteria* and *Firmicutes* were commonly found in the same gut (fly gut numbers 1, 2, 5, 14, 15, 17, 18, 19, 22, 23, 24 and 25). *Firmicutes* and *Proteobacteria* was jointly recorded in fly gut number 2 and 10. Only one fly gut number 2 had three different bacteria phylum and that *is Firmicutes, Proteobacteria* and *Bacteroidetes* (Fig. 1). 15 flies (20%) had a single occurrence of a bacterial phylum (See numbers 3, 4, 6, 7, 8, 9, 11, 12, 13, 15, 16, 20, 21, 25, 26, 27 in Fig. 1) pertaining to the *Firmicutes* which isolation resulted from experiments conducted under anaerobic conditions. The single dominant phylum isolated from fly guts in the presence of oxygen was *Firmicutes* from 9 flies out of 10 and only one with *Proteobacteria.*

### Impact of aeration on bacteria

Both aerobic and anaerobic techniques were used to ensure maximum harvesting of all bacteria found in the fly guts. However, the results showed that a wider diversity of bacteria was isolated under anaerobic conditions than under aerobic condition (Table 2). Under anaerobic conditions bacterial isolates were distributed within four phyla including *Firmicutes*, *Proteobacteria*, *Actinobacteria* and *Bacteroidetes;* whereas in the presence of oxygen, pure cultures pertained to only two phyla (*Firmicutes*, *Proteobacteria*). These results demonstrated that isolation under anaerobic conditions was more efficient probably mimicking the physico-chemical conditions existing in the fly gut as the latter is sealed from easy access to oxygen. *Firmicutes* and *Proteobacteria* bacteria were found using both isolation methods, thus suggesting that most of them should be considered as facultative anaerobes, while members of the *Bacteroidetes* and *Actinobacteria* bacterias, appear to be anaerobes. Despite the fact that the majority members of *Actinobacteria* are known to be aerobic, few of them, such as *Actinomyces israelii*, can grow under anaerobic conditions [15].

## Discussion

### The predicted role of isolated bacteria in relation to tsetse refractoriness

**Fig. 2:**
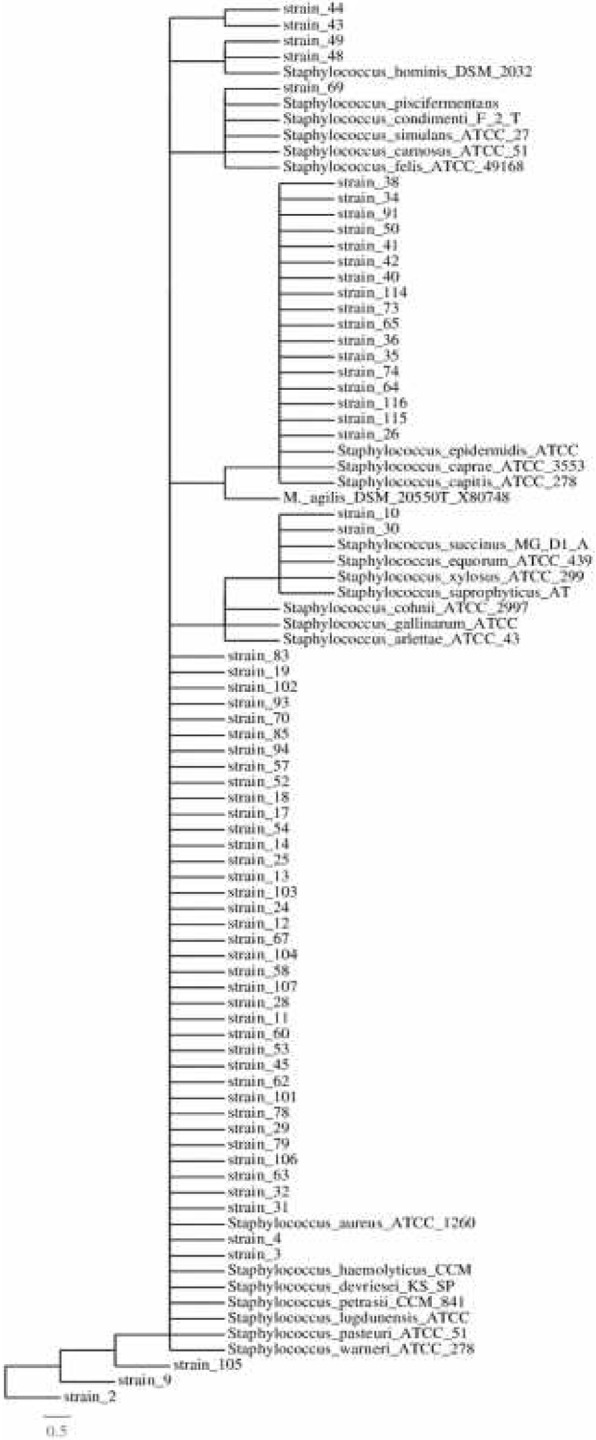
Maximum-likelihood phylogenetic tree based on the comparative analysis of 16S rRNA gene sequences showing the relationships between *Glossina* isolated bacterial strains and the respective other bacteria species: 16S rRNA-based tree reflecting the phylogenetic relationships of staphylococci strains isolated by culture of *G. pallidipes* midguts. The tree is based on a parsimony tree and a data set containing all available almost complete 16S rRNA sequences from isolated strains and selected reference of staphylococci as well as *Micrococcus agilis*. The tree topology was corrected according to the results of distance matrix as well as maximum-parsimony analyses (100 re-sampling). Visualisation of the tree was made with TreeDyn. The bar indicates estimated sequence divergence

In this study, we demonstrate through culture-dependent studies that tsetse midguts are inhabited by a wide diversity of bacteria. While in some guts only one bacterial phylum is represented, other guts contained bacteria pertainig to different phyla. Using anaerobic conditions, we succeeded in isolating microorganisms within the *Firmicutes*, the *Proteobacteria*, the *Actinobacteria* and the *Bacteroidetes*. In contrasts, under aerobic conditions of growth only members affiliated to the *Firmicutes and* the *Proteobacteria* were retrieved. All these isolates belonged to different genera which are reported in Table 2. Although the metabolical and ecological roles of some isolated bacteria have been reported before, there are no known roles. While all strains isolated have been reported in Fig. 2, the 11 major known species were placed as shown in various phylogenetic trees (Additional files 1, 2, 3, 4, 5,6, 7, 8 and 9). The predictive roles of these isolates are discussed below.

### Bacteria belonging to the FIRMICUTES (*Staphylococcus* spp.*, Bacillus* spp., *Vagococcus* spp.*)*

The most prevalent bacteria belonging to the *Firmicutes* comprised *Staphylococcus*, *Bacillus* and *Vagococcus* species. These are found worldwide and reside normally on the skin and mucous membranes of humans and other organisms.

### The genus *Staphylococcus*

In this study the majority of the *Staphylococcus* spp. were closely phylogenetically related (similarities of between 97 and 99%) to the following: *S. arlettae; S. aureus; S. capitis; S. caprae; S. carnosus; S. cohnii; S. condimenti; S. devriesei; S. epidermidis; S. equorum; S. gallinarum; S. haemolyticus; S. hominis; S. kloosii; S. lugdunensis; S. lutrae;; S. pasteuri; S. petrasii; S. piscifermentans; S. devriesei; S. saprophyticus; S. succinus; S. warneri; S. xylosus* with similarities of between 97 and 99%. *Staphylococcus* species are facultative anaerobes. They have previously been isolated in the gut of *Glossina palpalis palpalis* [10], *G.f. fuscipes* [5], and in malaria mosquitoes [16, 17]. *Staphylococcus* spp. are generally pathogenic bacteria causing various diseases in human by producing various factors that are defensive against the host immune system, adhesive to host tissues, and toxins that destroy host tissues [18]. In insects, bacteria belonging to this genus are prevalent in Lepidoptera of the families *Sphingidae* and *Noctuidae*, where they could contribute to digestion and development of the velvet bean caterpillar [19]. Some of *Staphylococcus* spp. have proteolytic activity which is suggestive of their potential role of minimizing the harmful consequences of protease inhibitors from some of this insect host plants, such as soybean [19]. *Staphylococcus* spp. are also widely present in other Lepidoptera, (*Hyles euphorbiae* [20]*,* in mosquitoes [21]; wood-feeding termite (*Reticulitermes flavipes* [22]; in *G. p. palpalis* [9]. Previous results demonstrated that a core microbial community exists in the gut in other pest insects (*Spodoptera littoralis* and *Helicover paarmigera)*, which may contribute to the insect physiology. On the other hand, however, insect physiology and food can significantly influence some bacterial species in the gut. In addition, *Staphylococcus* spp. from the gut might also serve as a reservoir of microorganisms for ever-changing environments [20]. Some *Staphylococcus* spp.have been isolated from the gut of house flies (*Musca domestica)* [23].

### The genus *Bacillus*

The second group consisted of *Bacillus* species. These are rod-shaped Gram-positive bacteria, which can be either be obligate aerobes, or facultative anaerobes (Table 1); they are important pathogens, causing anthrax and food poisoning. Both *Bacillus* and *Staphylococcus* spp. have been demonstrated to affect the survival of their insect hosts and or insect vector competence as reviewed extensively by Geiger et al.*,* [10]. *Bacillus* spp. have been isolated in the red fire ant [24]; in mosquitoes [17], *G. f. fuscipes* [5], and in house flies [25]. These bacteria within the *Firmicutes* phylum are also responsible for virulence factors [26]*.*

### The genera *Vagococcus* and *Enterococcus*

Other species within the *Firmicutes* were the *Vagococcus* and *Enterococcus* spp. and *Vagococcus* spp. that have been isolated from midgut of *Culex quinquefasciatus* mosquito and house flies [23] and green bottle flies [27]. The occurrence of *Enterococcus* spp. was noted in two midguts only and occurred in association with *Vagococcus* spp. These bacteria have been isolated from the gut of *G. p. palpalis* [4, 28]; red fire ant [24]; in *Anopheles stephensi* [17].

The roles of *Bacillus, Staphylococcus, Vagococcus* and *Enterococcus* spp. in tsetse flies (blood sucking insect) are unknown and need to be further examined to pave the way for developing novel pest control strategies.

### Bacteria belonging to the *PROTEOBACTERIA* (*Acinetobacter* spp.*; Mesorhizobium* spp.*; Paracoccus* spp.*; Psychrobacter* spp.*)*

Some members of this phylum are known as Gram-negative, aerobic and non-spore-forming bacteria whereas others are anaerobes. They have been documented to display antiparasitic activity in the guts of insects [29].

### The genus *Acinetobacter*

These are potentially pathogenic opportunistic bacteria. They have also been isolated from *G. p. palpalis* trapped from Angola and Cameroon [8, 10]. In this study they were isolated from *G.pallidipes* which is a savannah tsetse species. *Acinetobacter* spp. have also been reported to inhabit guts of several insect species where they may play various different roles including production of antiparasitic compounds and antiparasitic activity in the guts of insects [29]. They are known to be responsible for (i) complete development of *Stomoxyx calcitrans* fly larvae [30], (ii) hemolysins, antibiotics, and hemagglutinin activities as reviewed by Geiger et al.*,* [8]. These bacteria have also been reported to reside in the gut of *G. p. palpalis* and *G. pallicera* [10]. *Acinetobacter* spp. were also isolated from the gut of *Anopheles* mosquitoes [17]. *Low populations of Acinetobacter spp. were also recorded in G. pallidipes in Uganda* [31]*.*

### The genus *Mesorhizobium*

*Mesorhizobium* spp. are Gram-negative soil bacteria nitrogen-fixing species and mostly found on root nodules. In this respect, its role in the gut of blood feeding insect is not clear. However, besides root nodules, members of this genus have been encountered at several occasions in various ecosystems [32].

### The genus *Paracoccus*

*Paracoccus* spp.are Gram-negative bacteria found in either aerobic or anaerobic environments [33]. In this study they were only isolated under anaerobic conditions (Table 2). They are found in the environment as an industrial effluent [34]; as well as in Cayenne ticks [35]; however their role in the gut of blood feeding insect remains to be elucidated.

### The genus *Psychrobacter*

*Psychrobacter* spp. are cocci shaped aerobic bacteria but surprisingly we were able to find them also under anaerobic cultures thus suggesting that some members of this genus may also display a metabolism in the absence of oxygen to be discovered (Table 2). The closest phylogenetic relative of our isolate in this study was *Psychrobacter pulmonis* which was isolated from the lungs of the lamb [36]. Their exact role in blood sucking insects is unknown.

### Bacteria belonging to the phylum ACTINOBACTERIA *(Microbacterium* spp., *Micrococcus* spp., *Arthrobacter* spp., *Corynebacterium* spp.*, Curtobacterium* spp.*, Dietzia* spp*)*

*Actinobacteria* is a phylum of Gram-positive bacteria playing an important role to humans because agriculture and forests depend on their contributions to soil systems. In soil, they behave much like fungi, helping to decompose the organic matter of dead organisms so that the molecules can be taken up anew by plants. In this study the members of this phylum that were isolated were found using anaerobic culture conditions (Table 2). Some of them have been isolated from the gut of mosquitoes, e.g. *Anopheles gambiae* [16].

### The genus *Microbacterium*

These are Gram- positive bacteria; mostly aerobic; but weak anaerobic growth may occur in a wide range of environments including milk, dairy products, fresh beef, poultry raw sewage, soil, activated sludge, and human clinical specimens. Others are opportunistic pathogens. In this study, we were able to isolate bacteria from the tsetse fly gut having 100% similarity with *Microbacterium testaceum* and lower similarities with *Microbacterium xylanilyticum* (97%) *Microbacterium flavescens* (98%). These bacteria have also been documented to occur in the gut of larvae *Anopheles stephensi* [17].

### The genus *Micrococcus*

They are Gram-positive bacteria found in a wide range of environments, some of the members are generally regarded as harmless saprophytes (non pathogenic) that inhabit or contaminate the skin, mucosa, and perhaps also the oropharynx. However, they can be opportunistic pathogens for the immunocompromised individuals. On human skin, they convert sweat odorless compounds into sweat compounds with an unpleasant odor. These bacteria have also been documented to occur in the gut of adult *Anopheles stephensi* [17].

### The genus *Arthrobacter*

These are Gram-positive bacteria commonly found in the soil. They are known to degrade agricultural pesticides. Bacteria of the genus *Arthrobacter* are thought to play a significant role in many ecosystems and affect human welfare; they have been isolated from the gut of Sub-cortical Beetle (*Agrilus planipennis*) [37]. However, the association of these bacteria with the gut of tsetse flies has not been described so far.

### The genus Corynebacterium

*Corynebacterium* spp. are Gram-positive and aerobic rod-shaped; widely distributed in nature in the microbiota of animals (including the human microbiota) and are mostly innocuous. They are found in the mucosa and normal skin flora of humans and animals with some species being known for their pathogenic effects in humans and other animals [38]. These bacteria have also been documented to occur in the gut of adult and larvae of *Anopheles gambiae* [17]. *Low populations of these bacteria were also recorded in G. pallidipes from Uganda* [31]*.* Their role in the gut of a blood sucking insect and especially tsetse flies is unknown.

### The genus *Curtobacterium*

These are Gram-positive microorganisms which have been recovered from soils andcause bacterial wilt in some plants, especially beans [39]. They have been isolated in the gut of Sub cortical Beetle (*Agrilus planipennis*) [37].

### The genus *Dietzia*

*Dietzia* spp. are aerobic, Gram-positive bacteria with some of the members being found in various environments including soil, deep sea sediment, soda lakes, and marine aquatic life and from the gut of pupae of the obligate parasitic fly, *Wohlfahrtia magnifica* [40]. The dipterous larvae of this insect are obligate parasites of living warm-blooded vertebrates causing myiasis in most domesticated animals and an infestation of live and/or dead organs and tissues of vertebrates. The bacteria from this group have been isolated from the gut of the larvae of the Japanese Horned Beetle (*T. dichotomus*) and some are potential carrier by zoonotic and arthropod vectors [41]. They have been isolated from the gut of *Aedes albopictus* hence implicated as a suitable candidate for paratransgenesis [42]. However, the role of in blood sucking insects is still unknown hence further investigation is required.

## BACTEROIDETES (*Flavobacterium* spp.)

### The genus *Flavobacterium*

In this study, the *Flavibacterium* isolates were facultative anaerobic bacteria and their prevalence was very low. They are widely distributed in soils, sediments, and sea water, as well as in the guts and on the skin of animals. They represent the second most abundant microbiota in the human gut [43]. Their role in the gut of bloodsucking insects is not clear but it has been documented by Franca et al, [44] as one of the bacterial types that were found contaminating blood units. These bacteria have been reported in the gut of *G. p. palpalis* [11] and in malaria mosquitoes *Anopheles gambie* [16] which were collected in Cameroon.

## Conclusion

This study based on culure-dependent approaches reveals that the gut of tsetse fly possesses a rich bacterial diversity encompassing a wide range of phyla within the domain *Bacteria*. Yun et al [45] reported that the relative bacterial abundance in the gut varies according to the environmental habitats of the insect and is also associated with thein situ level of oxygen. Bacterial diversity is known to be higher in omnivorous insects than stenophagous (carnivores and herbivores) ones. Hence the bacterial diversity in insects may be related to the food types consumed. Further research is thus recommended in order to unravel their role in epidemiology of African Trypanosomiasis and to develop potential new anti-vector strategies to definitively eliminate this deadly disease, which is the goal for the future years.

The majority of the bacteria isolated from *G. pallidipes* midgut isolated either under aerobic or anaerobic conditions have already been found to be associated to insects in general and in tsetse flies in particular. In this respect, we expect them to play a significant ecological role in the digestive tract of the latter. However, such hypothesis probably needs further investigation to be validated. This role might be linked to defense mechanism against harmful parasites contained in blood-meals or encountered on the skin surface of a host when piercing to obtain a blood meal. Other predictive roles could be blood degradation and assisting in digestive processes of blood meal and other essential activities related to fly survival.

Further studies are necessary to know if any of the isolates that we obtained may or not favor the establishment of the parasites in the flies and hence could be useful in the modulation of sleeping sickness disease and also play a significant party in vector control.

In this study we have managed to cultivate bacteria which point to the importance of metagenomic analysis to analyze microbial diversity and dynamics by studying the genomic content of the microbiota; coupled with metataxonomic analysis of analyzing high-throughput sequencing data, primarily from 16S rRNA gene sequencing and DNAseq, to identify microorganisms and viruses within a complex mixture [46]. In this respect, there is clearly a need to characterize these microorganisms and also others that we have isolated from the tsetse fly’s gut to provide evidence of their metabolic features and therefore understand the ecological role that they may play in situ. We may expect also from such studies to have the opportunity to describe novel bacteria at the species or genus level.

### Phylogenetic trees of isolated bacteria

*Microbacterium* spp. appear as strains 46, 47, 51 and 68; *Micrococcus* spp. as strain 66; *Paracoccus* spp. as strain 27; Vagococcus spp. strains 108, 110; Acinetobacter spp. *as* strain 5; Arthrobacter spp. *as* strains 61 and 84; *Bacillus spp as* strains 16 and 56; *Curtobacterium* spp. *as* strain 1; *Dietza* spp. as strain 15 and *Mesorhizobium* spp. as strain 100 (individual trees of isolated species will appear as Additional files 1, 2, 3, 4, 5,6, 7, 8, 9 and 10). However, all these are included in the phylogenetic tree Fig. 2.

## Additional files

Additional file 1: Maximum-likelihood phylogenetic tree based on the comparative analysis of 16S rRNA gene sequences. Phylogenetic position of strains 46, 47, 51 and 68 within the genus *Microbacterium* spp., *Arthrobacter globiformis* (M23411) was used as the out-group. Bootstrap values (1000 tree replications) higher than 60% are indicated at the nodes of the tree. (PDF 44 kb)

Additional file 2: Maximum-likelihood phylogenetic tree based on the comparative analysis of 16S rRNA gene sequences. *Glossina pallidipes* isolated bacterial strain 66 was *Micrococcus* spp. and the sequence of *Cellulomonas flavigena* (DSM 20109 T X83799) was used as the out-group. Bootstrap values (1000 tree replications) higher than 60% are indicated at the nodes of the tree. (PDF 49 kb)

Additional file 3: Maximum-likelihood phylogenetic tree based on the comparative analysis of 16S rRNA gene sequences. *Glossina pallidipes* isolated bacterial strain 27 was *Paracoccus* spp. and the sequence of *Roseobacter denitificans* (OCh 114 T M96746) was used as the out-group. Bootstrap values (1000 tree replications) higher than 60% are indicated at the nodes of the tree. (PDF 54 kb)

Additional file 4: Maximum-likelihood phylogenetic tree based on the comparative analysis of 16S rRNA gene sequences. *Glossina pallidipes* isolated bacterial strains 108, 110 were *Vagococcus* spp. and the sequence of *Streptococcus lactis* (ATCC 19435 T M58837) was used as the out-group. Bootstrap values (1000 tree replications) higher than 60% are indicated at the nodes of the tree. (PDF 34 kb)

Additional file 5: Maximum-likelihood phylogenetic tree based on the comparative analysis of 16S rRNA gene sequences. *Glossina pallidipes* isolated bacterial strain 5 were *Acinetobacter* spp.and the sequence of *Moraxella lacunata* (ATCC 17967 T AF005160) and *Psychrobacter immobilis* (ATCC 43116 T U39399) were used as out groups. Bootstrap values (1000 tree replications) higher than 60% are indicated at the nodes of the tree. (PDF 53 kb)

Additional file 6: Maximum-likelihood phylogenetic tree based on the comparative analysis of 16S rRNA gene sequences. *Glossina pallidipes* isolated bacterial strains 61 and 84 were *Arthrobacter* spp., and the sequence of *Mycobacterium smegmatis* (ATCC 19420 T AY457078) was used as the out-group. Bootstrap values (1000 tree replications) higher than 60% are indicated at the nodes of the tree. (PDF 44 kb)

Additional file 7: Maximum-likelihood phylogenetic tree based on the comparative analysis of 16S rRNA gene sequences. *Glossina pallidipes* isolated bacterial strains 16 and 56 were *Bacillus* spp., and the sequence of *Paenibacillus polymyxa* (IAM 13419 T D16276) was used as the out-group. Bootstrap values (1000 tree replications) higher than 60% are indicated at the nodes of the tree. (PDF 37 kb)

Additional file 8: Maximum-likelihood phylogenetic tree based on the comparative analysis of 16S rRNA gene sequences. *Glossina pallidipes* isolated bacterial strain 1 was *Curtobacterium* spp., and the sequence of *Tsukamurella paurometabolum* (X53207) was used as the out-group. Bootstrap values (1000 tree replications) higher than 60% are indicated at the nodes of the tree. (PDF 51 kb)

Additional file 9: Maximum-likelihood phylogenetic tree based on the comparative analysis of 16S rRNA gene sequences. *Glossina pallidipes* isolated bacterial strain 15 was *Dietza*spp.and the sequence of *Arthrobacter globiformis* (DSM 20124 T X80736) was used as the out-group. Bootstrap values (1000 tree replications) higher than 60% are indicated at the nodes of the tree (PDF 42 kb)

Additional file 10: Maximum-likelihood phylogenetic tree based on the comparative analysis of 16S rRNA gene sequences. *Glossina pallidipes* isolated bacterial strain 100 was *Mesorhizobium* spp., and the sequences of *Azorhizobium caulinodans* (ORS 571 T D11342) and *Bradyrhizobium japonicum* (LMG 6138 T X66024) were used as the out-group. Bootstrap values (1000 tree replications) higher than 60% are indicated at the nodes of the tree (PDF 36 kb)

## Abbreviations

AT: African Trypanosomiasis
CO_2_: Carbon dioxide
HAT: Human African Trypanosomiasis
NCBI: National Center for Biotechnology Information
PCR: Polymerase Chain Reaction
