# Supplementary figures and images for: Bacterial diversity obtained by culturable approaches in the gut of Glossina pallidipes population from a non sleeping sickness focus in Tanzania: preliminary results
Source: BMC Microbiol. 2018 Nov 23;18(Suppl 1):164. doi: 10.1186/s12866-018-1288-3 (PMC6251091; doi:10.1186/s12866-018-1288-3)

Figure 3

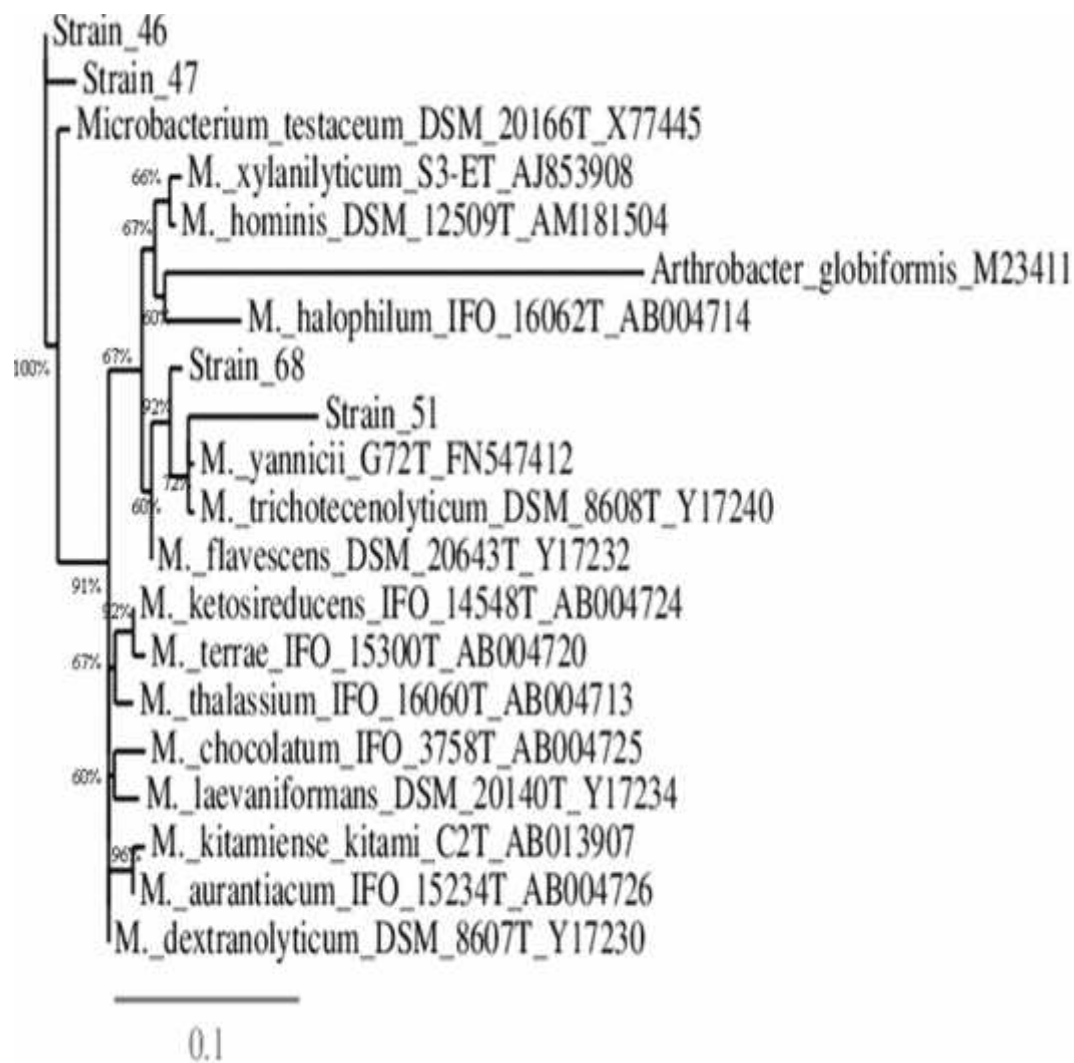

Supplement: Supplementary file 1 — Maximum-likelihood phylogenetic tree based on the comparative analysis of 16S rRNA gene sequences. Phylogenetic position of strains 46, 47, 51 and 68 within the genus Microbacterium spp., Arthrobacter globiformis (M23411) was used as the out-group. Bootstrap values (1000 tree replications) higher than 60% are indicated at the nodes of the tree. (PDF 44 kb) [file 12866_2018_1288_MOESM1_ESM.pdf]

**Figure 4**

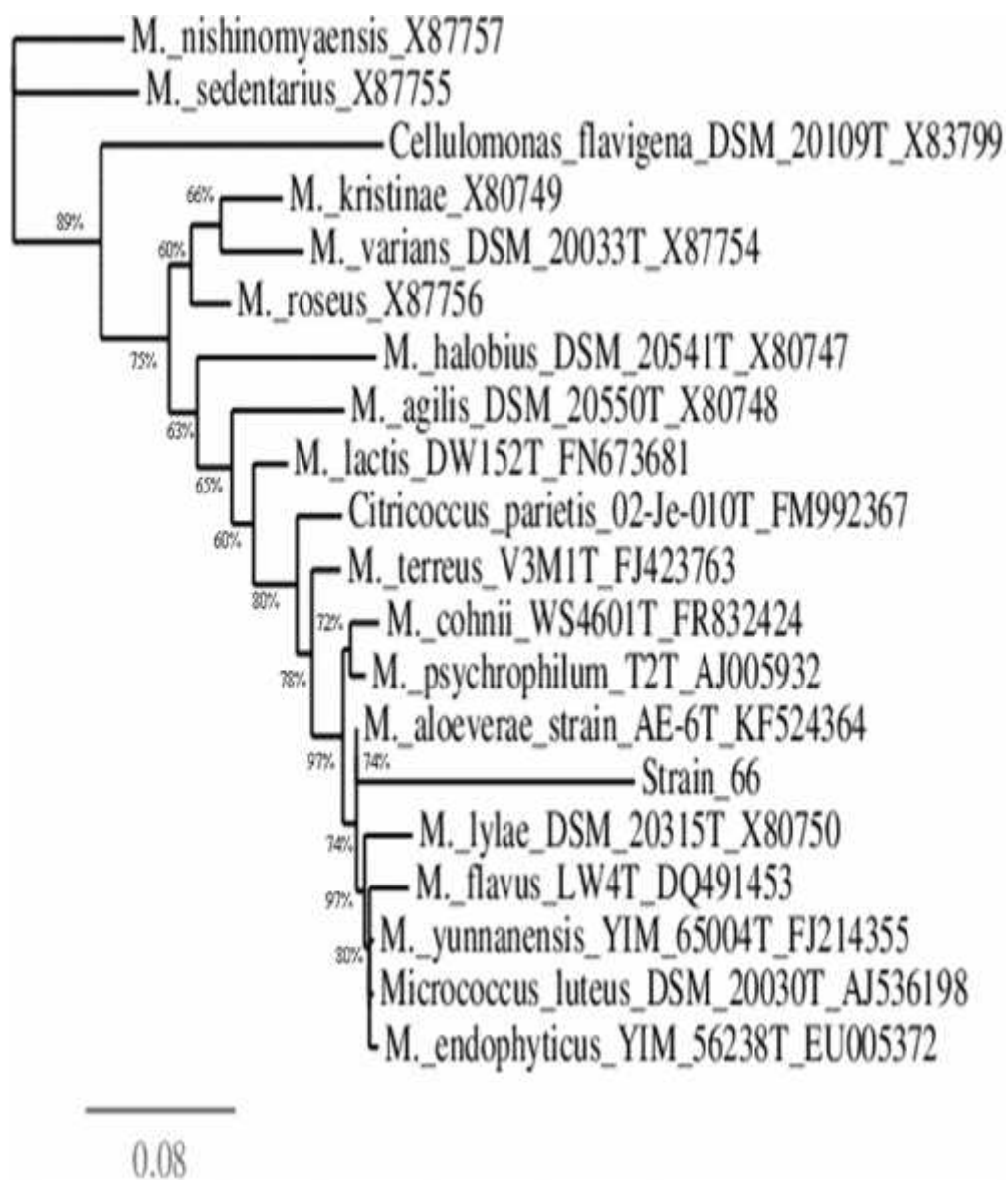

Supplement: Supplementary file 2 — Maximum-likelihood phylogenetic tree based on the comparative analysis of 16S rRNA gene sequences. Glossina pallidipes isolated bacterial strain 66 was Micrococcus spp. and the sequence of Cellulomonas flavigena (DSM 20109 T X83799) was used as the out-group. Bootstrap values (1000 tree replications) higher than 60% are indicated at the nodes of the tree. (PDF 49 kb) [file 12866_2018_1288_MOESM2_ESM.pdf]

Figure 5

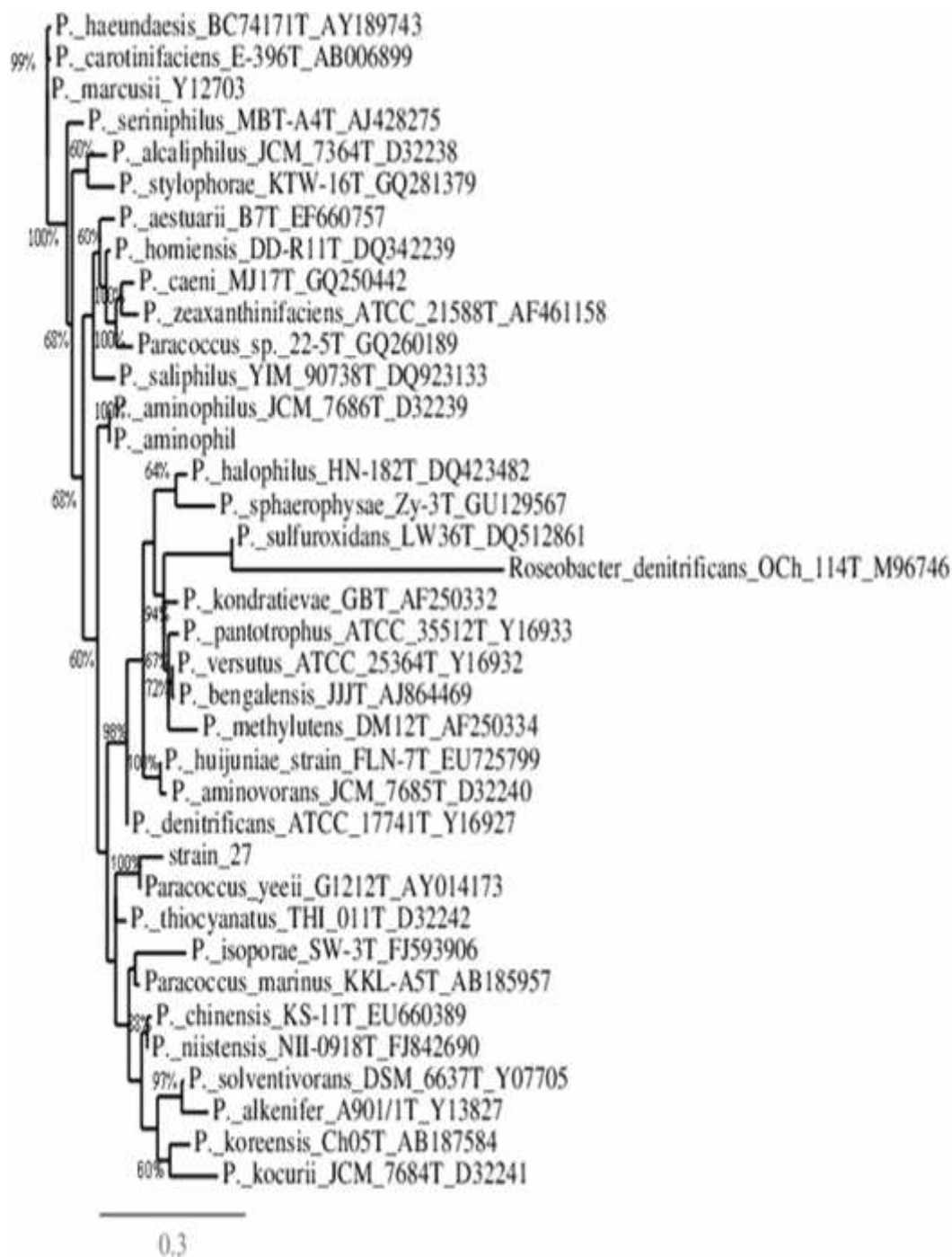

Supplement: Supplementary file 3 — Maximum-likelihood phylogenetic tree based on the comparative analysis of 16S rRNA gene sequences. Glossina pallidipes isolated bacterial strain 27 was Paracoccus spp. and the sequence of Roseobacter denitificans (OCh 114 T M96746) was used as the out-group. Bootstrap values (1000 tree replications) higher than 60% are indicated at the nodes of the tree. (PDF 54 kb) [file 12866_2018_1288_MOESM3_ESM.pdf]

**Figure 6**

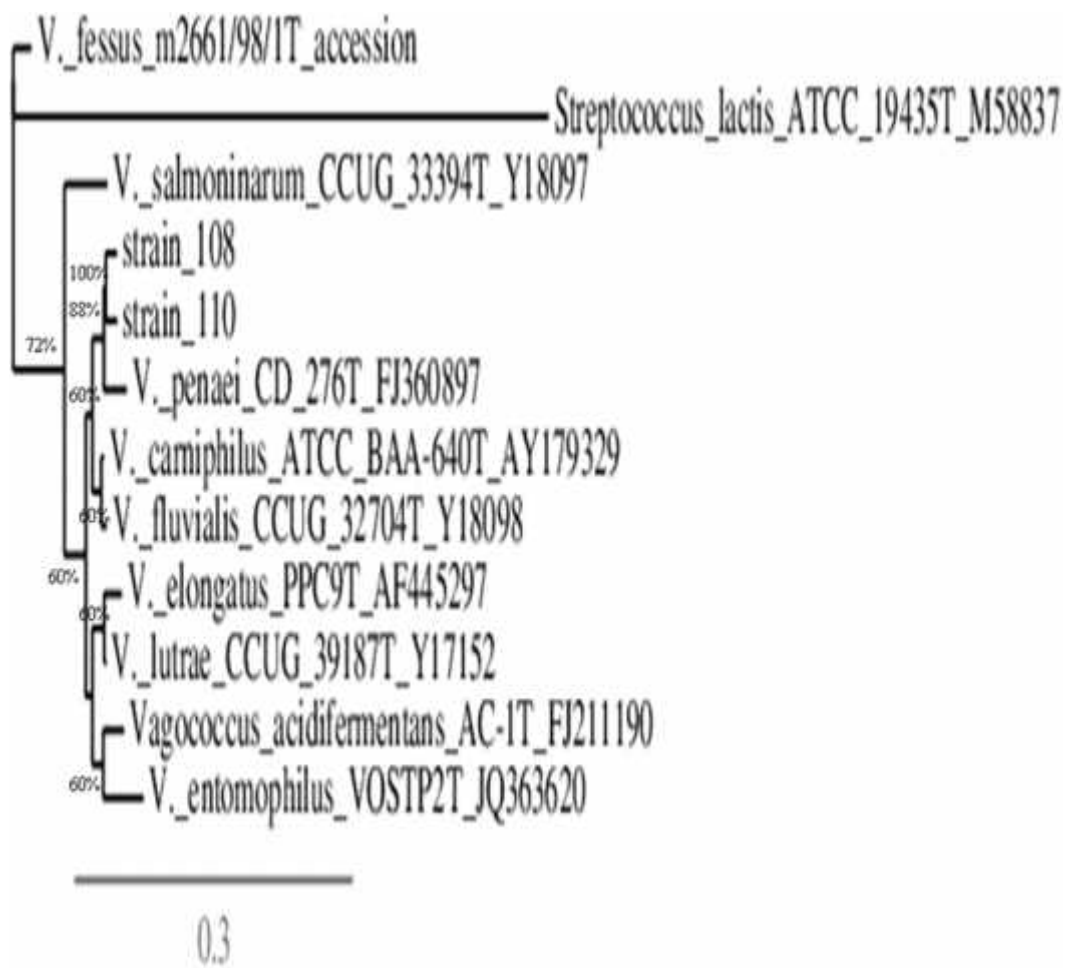

Supplement: Supplementary file 4 — Maximum-likelihood phylogenetic tree based on the comparative analysis of 16S rRNA gene sequences. Glossina pallidipes isolated bacterial strains 108, 110 were Vagococcus spp. and the sequence of Streptococcus lactis (ATCC 19435 T M58837) was used as the out-group. Bootstrap values (1000 tree replications) higher than 60% are indicated at the nodes of the tree. (PDF 34 kb) [file 12866_2018_1288_MOESM4_ESM.pdf]

**Figure 7**

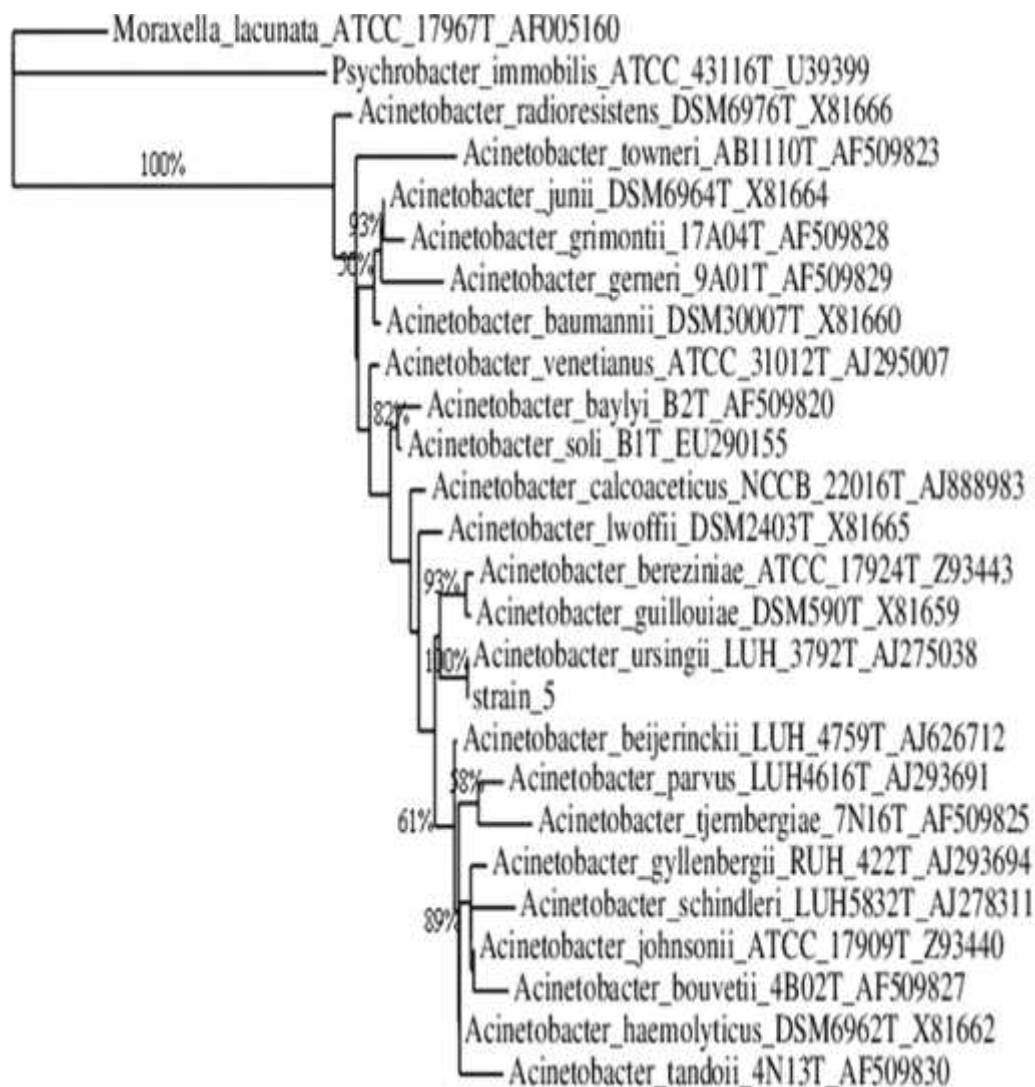

Supplement: Supplementary file 5 — Maximum-likelihood phylogenetic tree based on the comparative analysis of 16S rRNA gene sequences. Glossina pallidipes isolated bacterial strain 5 were Acinetobacter spp.and the sequence of Moraxella lacunata (ATCC 17967 T AF005160) and Psychrobacter immobilis (ATCC 43116 T U39399) were used as out groups. Bootstrap values (1000 tree replications) higher than 60% are indicated at the nodes of the tree. (PDF 53 kb) [file 12866_2018_1288_MOESM5_ESM.pdf]

Figure 8

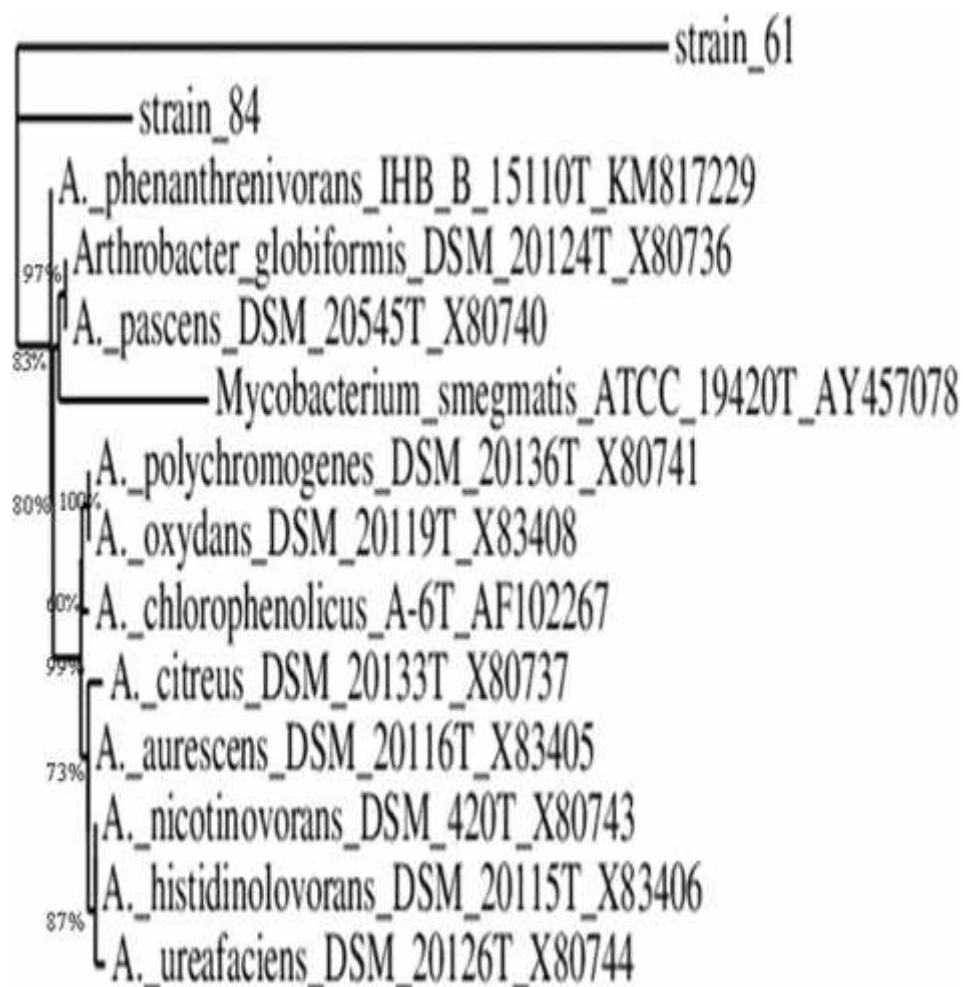

Supplement: Supplementary file 6 — Maximum-likelihood phylogenetic tree based on the comparative analysis of 16S rRNA gene sequences. Glossina pallidipes isolated bacterial strains 61 and 84 were Arthrobacter spp., and the sequence of Mycobacterium smegmatis (ATCC 19420 T AY457078) was used as the out-group. Bootstrap values (1000 tree replications) higher than 60% are indicated at the nodes of the tree. (PDF 44 kb) [file 12866_2018_1288_MOESM6_ESM.pdf]

**Figure 9**

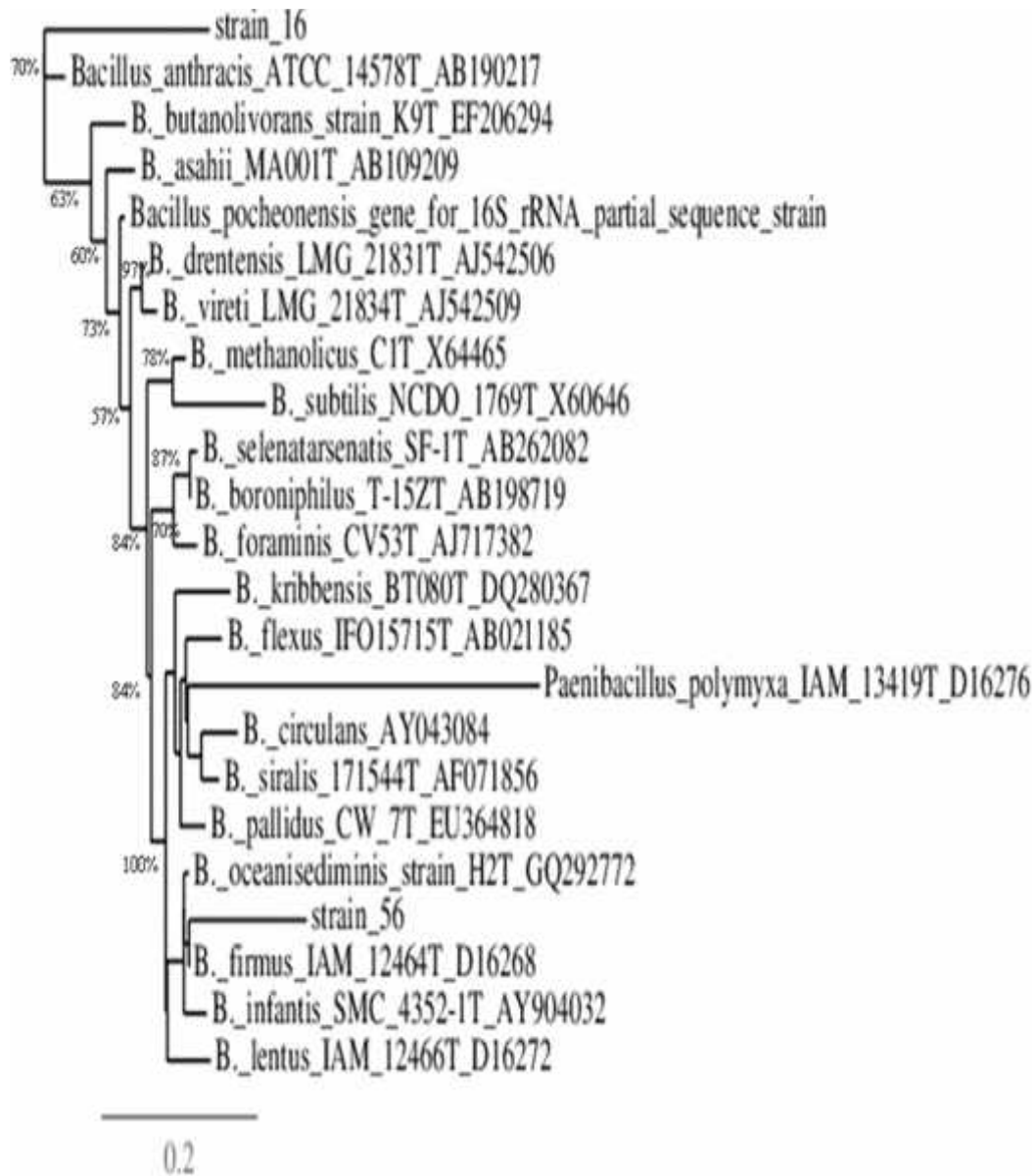

Supplement: Supplementary file 7 — Maximum-likelihood phylogenetic tree based on the comparative analysis of 16S rRNA gene sequences. Glossina pallidipes isolated bacterial strains 16 and 56 were Bacillus spp., and the sequence of Paenibacillus polymyxa (IAM 13419 T D16276) was used as the out-group. Bootstrap values (1000 tree replications) higher than 60% are indicated at the nodes of the tree. (PDF 37 kb) [file 12866_2018_1288_MOESM7_ESM.pdf]

**Figure 10**

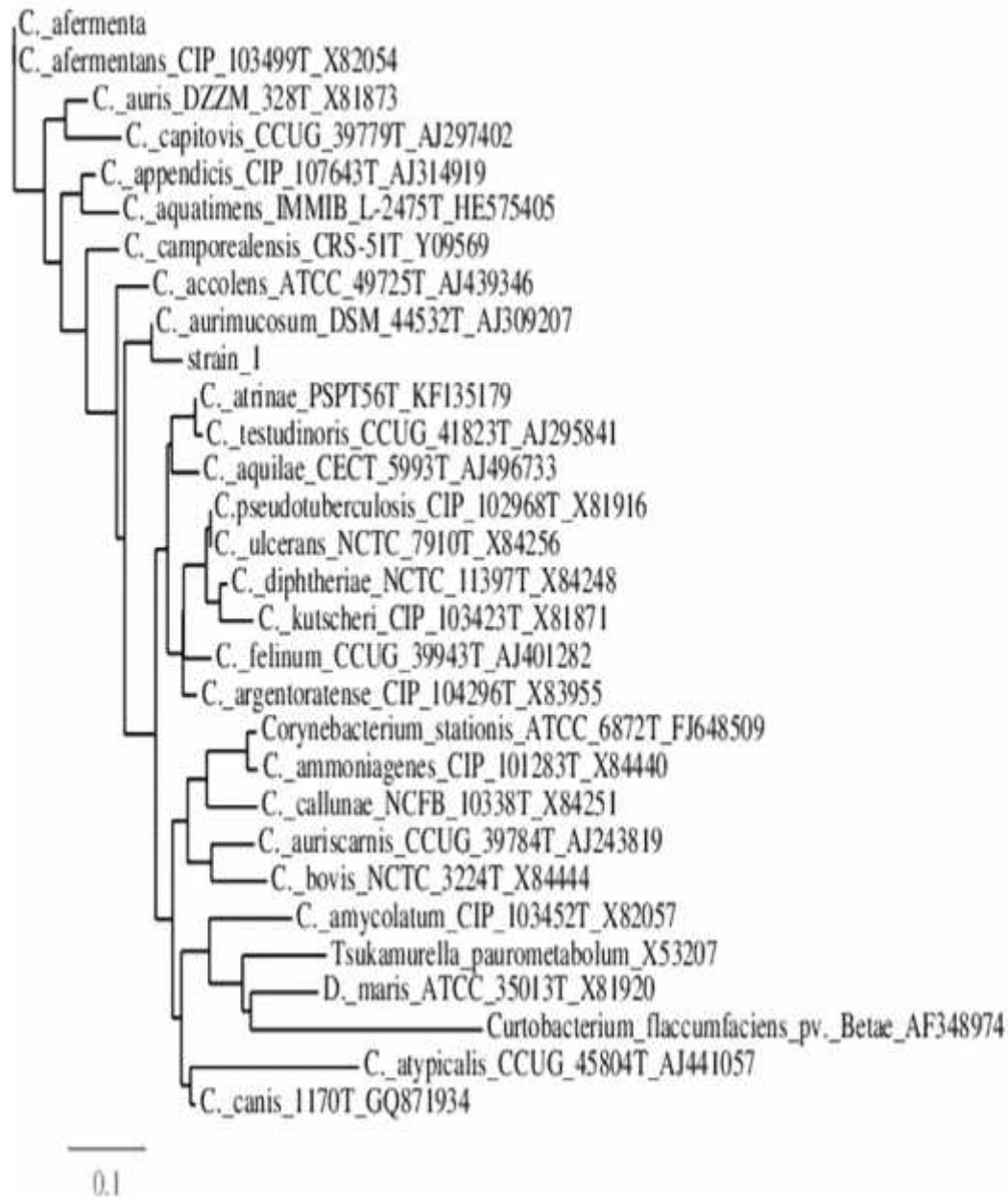

Supplement: Supplementary file 8 — Maximum-likelihood phylogenetic tree based on the comparative analysis of 16S rRNA gene sequences. Glossina pallidipes isolated bacterial strain 1 was Curtobacterium spp., and the sequence of Tsukamurella paurometabolum (X53207) was used as the out-group. Bootstrap values (1000 tree replications) higher than 60% are indicated at the nodes of the tree. (PDF 51 kb) [file 12866_2018_1288_MOESM8_ESM.pdf]

**Figure 11**

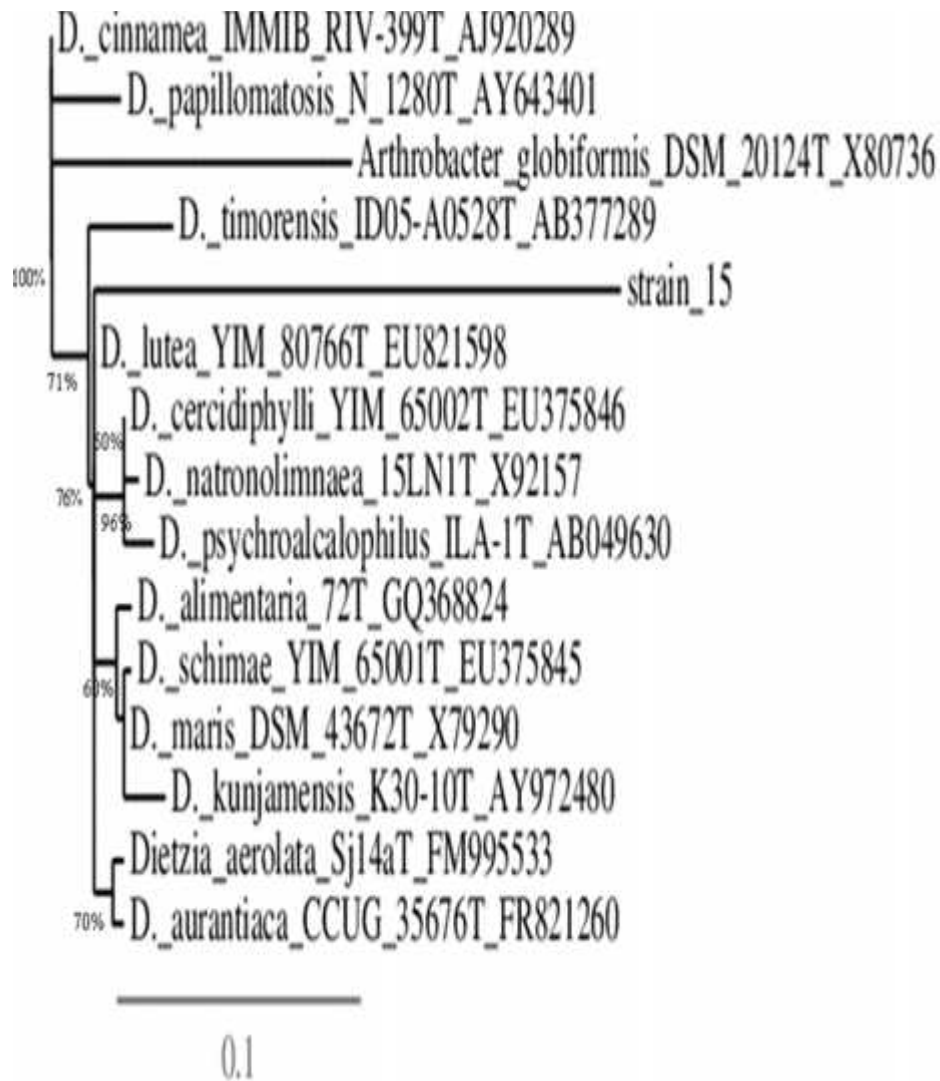

Supplement: Supplementary file 9 — Maximum-likelihood phylogenetic tree based on the comparative analysis of 16S rRNA gene sequences. Glossina pallidipes isolated bacterial strain 15 was Dietzaspp.and the sequence of Arthrobacter globiformis (DSM 20124 T X80736) was used as the out-group. Bootstrap values (1000 tree replications) higher than 60% are indicated at the nodes of the tree (PDF 42 kb) [file 12866_2018_1288_MOESM9_ESM.pdf]

Figure 12

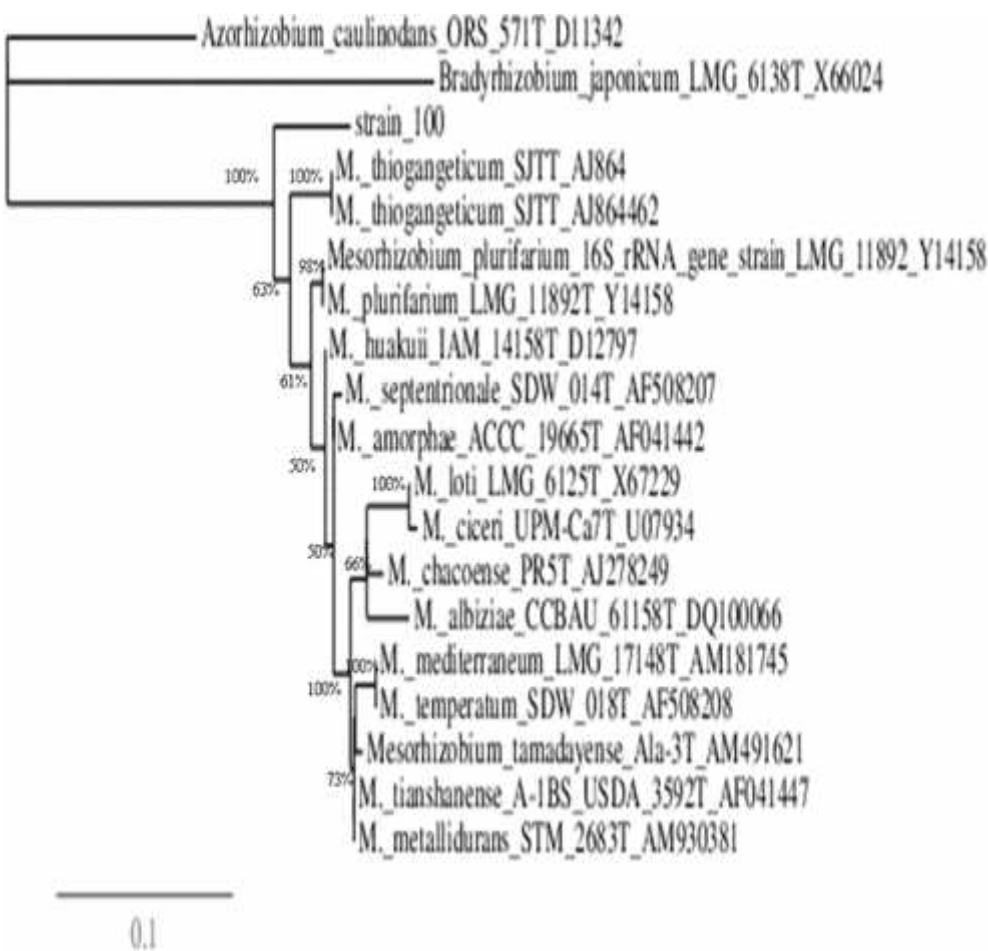

Supplement: Supplementary file 10 — Maximum-likelihood phylogenetic tree based on the comparative analysis of 16S rRNA gene sequences. Glossina pallidipes isolated bacterial strain 100 was Mesorhizobium spp., and the sequences of Azorhizobium caulinodans (ORS 571 T D11342) and Bradyrhizobium japonicum (LMG 6138 T X66024) were used as the out-group. Bootstrap values (1000 tree replications) higher than 60% are indicated at the nodes of the tree (PDF 36 kb) [file 12866_2018_1288_MOESM10_ESM.pdf]
